# Comparison of low temperature thermoplastic and 3D printed (TPU and PLA) CMC joint stabilization orthoses in healthy participants

**DOI:** 10.1038/s41598-026-39208-w

**Published:** 2026-02-11

**Authors:** Enver Güven, Ali Koray Özgün, Serap Alsancak

**Affiliations:** Department of Prosthetics & Orthotics, Faculty of Health Sciences, Ankara University, Ankara, Turkey

**Keywords:** CMC orthosis, 3D orthosis, Thermoplastic orthosis, Low temperature thermoplastic, Carpometacarpal joint, Hand orthosis, Orthopaedics, Rehabilitation

## Abstract

Carpometacarpal (CMC) osteoarthritis (OA) significantly impacts hand function and quality of life, particularly in older adults, and highlights the importance of effective conservative management strategies. Orthoses are a preferred conservative treatment, with recent advancements in 3D printing offering personalized alternatives. However, data comparing the outcomes of 3D-printed orthoses and traditional thermoplastic options are limited. Evaluating orthosis materials under controlled conditions may provide preliminary insights relevant for future clinical research. This study aims to evaluate and compare user satisfaction and functional outcomes of CMC orthoses made from thermoplastic polyurethane (TPU), polylactic acid (PLA), and low temperature thermoplastic (LTT) materials in healthy individuals. A comparative analysis conducted on healthy volunteers to assess satisfaction and functionality of different orthosis materials. Thirty healthy participants (15 males, 15 females, mean age 21 years) were recruited. Each participant used three types of CMC orthoses (TPU, PLA, and LTT) for two hours. User satisfaction was assessed using the Quebec Assistive Technology User Satisfaction Evaluation (QUEST 2.0), and hand function was measured using the Jebsen Taylor Hand Function Test (JTT). Statistical analyses appropriate for repeated-measures designs were used for group comparisons. TPU and LTT orthoses achieved statistically significant higher total scores on the QUEST 2.0 survey compared to PLA orthoses (*p* < 0.05). However, no statistically significant differences were observed between TPU and LTT orthoses. In the JTT total score, TPU orthoses performed statistically significantly better than both PLA and LTT orthoses (*p* < 0.05). No statistically significant differences were found between PLA and LTT orthoses. TPU orthoses demonstrated higher satisfaction scores compared to PLA and similar outcomes to LTT under short-term experimental conditions in healthy participants. These findings provide preliminary, experimental data obtained in healthy participants and may inform future clinical studies evaluating orthosis materials in symptomatic CMC osteoarthritis. Further studies in symptomatic populations with longer follow-up periods are needed to determine clinical relevance.

## Introduction

The carpometacarpal (CMC) joint is a saddle (sellar) articulation that allows the thumb to move in ways that are unique to it, such as opposition, pinch, and grasp. While this mobility is essential for hand function, it inherently compromises joint stability. As a result, the CMC joint is particularly susceptible to mechanical overload and degenerative change when stabilizing structures fail^1–3^. Biomechanical research has shown that during key-pinch tasks, dorsoradial bending moments at the base of the first metacarpal can reach levels that are several times higher than the force applied by the fingertip. This can cause higher joint contact pressures, which can lead to degeneration and instability^1,4^.

Due to these biomechanical traits, carpometacarpal osteoarthritis (CMC OA) ranks among the most common degenerative disorders of the hand, especially in those aged 50 and above. Around 22% of the general population experience symptomatic CMC OA, characterized by pain, inflammation, joint instability, and diminished hand function^5^. These symptoms can substantially reduce independence in daily activities and overall quality of life.

Surgical interventions, including trapeziectomy, ligament reconstruction, and tendon interposition, may be efficacious in advanced stages of disease; however, surgery is predominantly regarded as a last-resort option^6–8^. Thus, conservative management continues to be the fundamental approach to treatment for the majority of individuals with CMC OA. Among conservative treatments, CMC joint orthoses are commonly recommended to enhance joint alignment, restrict excessive movement, decrease mechanical loading, and mitigate pain^9,10^.

The clinical effectiveness of CMC orthoses has been supported by systematic reviews and meta-analyses demonstrating significant reductions in pain and improvements in hand function compared with placebo or no intervention^5,11^. Traditionally, these orthoses are fabricated using low temperature thermoplastic (LTT) materials molded directly on the patient’s hand. While clinically established, this method is highly dependent on practitioner skill and offers limited reproducibility.

Recent advances in three dimensional (3D) printing technology have introduced new possibilities for orthosis fabrication through digital scanning and computer-aided design. This approach enables highly customized orthoses with consistent geometry and potentially improved fit^12,13^. Among the materials commonly used in fused deposition modeling (FDM) 3D printing, thermoplastic polyurethane (TPU) and polylactic acid (PLA) differ markedly in mechanical behavior, with TPU offering flexibility and elastic recovery, and PLA providing rigidity and shape stability^14,15^. These differences may have important implications for joint stabilization, comfort, and functional performance in CMC orthoses.

While there is increasing interest in 3D-printed hand orthoses, the current literature mainly contrasts 3D-printed devices with traditional thermoplastic orthoses, rather than directly assessing various printing materials. To date, no studies have systematically compared different 3D printing filament materials with conventional LTT in the context of CMC joint stabilization orthoses. Unlike previous studies that primarily compared 3D printed orthoses with conventional thermoplastic devices, the present study directly compares different 3D printing filament materials (TPU and PLA) with LTT orthoses under controlled experimental conditions. By isolating material related effects in healthy participants, this study provides novel preliminary insights into how orthosis material properties influence user satisfaction and hand function. These findings contribute to informed material selection and may guide the design of future clinical studies in symptomatic CMC osteoarthritis populations.

Furthermore, isolating the effects of orthosis material in symptomatic CMC OA populations is challenging due to confounding factors such as pain severity, joint deformity, and disease stage. Therefore, the use of healthy participants provides a controlled experimental model that allows evaluation of material-related differences in comfort and functional performance without the influence of pathology-related variability.

Accordingly, the aim of this study was to compare LTT, TPU, and PLA CMC joint stabilization orthoses in terms of user satisfaction and hand function in healthy participants. This study is intended as a preliminary investigation to inform material selection and guide future clinical studies in individuals with symptomatic CMC osteoarthritis.

## Material & methods

This study evaluated satisfaction and functional outcomes in healthy individuals using three different CMC orthoses. Ethical approval was obtained from the Ankara University Institutional Research Ethics Board (Submission Number: 2023/621). All experiments were conducted in accordance with relevant institutional and national guidelines and regulations. Participants were recruited between December 2023 and February 2024. All participants received detailed information about the study and provided written informed consent prior to enrollment.

### Inclusion and exclusion criteria

Participants were eligible for inclusion if they were between 18 and 30 years of age and had no history of orthopedic, neurological, or rheumatological conditions affecting the upper extremities. All participants reported no pain or functional limitation in the thumb or hand and had no prior experience with thumb or CMC orthoses. Exclusion criteria included any history of hand or wrist surgery, current or previous diagnosis of carpometacarpal osteoarthritis, presence of systemic disease affecting musculoskeletal function, or acute upper extremity injury within the previous six months.

The study was conducted on 30 (15 female, 15 male) healthy individuals. CMC joint orthoses were made from 3 different materials for each individual. Once 3 orthoses were produced, individuals used each for 2 h. The order in which the orthoses were tested was randomized for each participant to minimize potential order and learning effects. Orthoses were designed as metagrip Orthoses to rest the CMC joint and limit joint movement^16^. Orthoses were made for each individual’s dominant extremity. 1st orthosis was made of LTT material in such a way that the CMC joint was in opposition and extension. For the LTT orthosis, a 2.6 mm thick thermoplastic sheet was heated in a water bath at approximately 65 °C until malleable. The softened material was then directly molded on the participant’s hand to achieve the standardized CMC joint stabilization design. After cooling, the orthosis retained its final shape, and edges were trimmed and smoothed for comfort. 3D scanning was performed for the 2nd and 3rd Orthois (with the Structure (Mark1- ST01) 3D scanner placed on the iPad (5th Generation). After the scanning data was received, it was transferred to the computer in stl format and from there to the Mashmixer program and the same design was made on the software.

The 3D-printed orthoses were manufactured using fused deposition modeling (FDM) technology with a Creality CR10-Max 3D printer. TPU orthoses were printed using flexible TPU filament (Shore hardness 75 A) with a nozzle temperature of 230 °C, build plate temperature of 60 °C, and a printing speed of 50 mm/s. PLA orthoses were printed using standard PLA filament with a nozzle temperature of 195 °C, build plate temperature of 50 °C, and a printing speed of 60 mm/s. For both TPU and PLA orthoses, a uniform orthosis thickness of 2.4 mm was used, with a layer height of 0.4 mm and 100% infill density to ensure consistent structural properties across materials. PLA in the 2nd orthosis and TPU in the 3rd orthosis were used as the printing material. The printing time of an orthosis on a 3D printer was 80 min.

Each orthosis was worn for a standardized period of two hours, during which participants were allowed to perform routine daily activities. Immediately following each wear period, user satisfaction was assessed using the Quebec Assistive Technology User Satisfaction Evaluation (QUEST 2.0), and hand function was evaluated using the Jebsen Taylor Hand Function Test (JTT). A standardized rest period of 30 min was provided between orthosis conditions to minimize potential carryover effects. The QUEST 2.0 instrument comprises 12 items, with eight focusing on the characteristics of the assistive device, such as dimensions (size), weight, adjustability, safety, durability, ease of use, comfort, and effectiveness. The remaining four items assess service factors, including service delivery, repairs and maintenance, professionalism, and follow-up support. The instrument also uses a 5-point scale to evaluate overall satisfaction with the device^17^.

Jebsen Taylor Hand Function test evaluates the speed at which individuals can perform seven specific tasks that reflect daily living activities, including writing a sentence, simulated page turning, and picking up small and large objects^18^. Before orthosis testing, all participants performed the Jebsen Taylor Hand Function Test without any orthosis to familiarize them with the tasks and reduce potential learning effects.

Statistical analysis: Descriptive statistics for continuous variables were reported as mean, standard deviation, median, minimum, and maximum values. Categorical variables were presented as counts and percentages. The normality of continuous variables was assessed using the Shapiro-Wilk test. For comparisons of scale scores among TPU, PLA, and LTT orthoses, repeated measures ANOVA was used for normally distributed data, with post hoc analyses conducted using Bonferroni tests to identify specific differences. For non-normally distributed data, the Friedman test was employed. When significant differences were detected, post hoc pairwise comparisons were performed using the Wilcoxon signed-rank test with appropriate adjustment for multiple comparisons. Statistical analyses were performed using IBM SPSS for Windows 20.0 (SPSS Inc., Chicago, IL), with a significance level set at *p* < 0.05. Effect sizes were calculated for repeated-measures analyses, and Kendall’s W was reported for Friedman tests. Given the exploratory nature of this study, a formal a priori sample size calculation was not performed. However, a post hoc power analysis based on the primary outcome measures (QUEST 2.0 total score and JTT total scores) indicated that a sample size of 30 participants was sufficient to detect moderate within-subject effects (effect size f = 0.25) with a statistical power exceeding 0.80 at an alpha level of 0.05. This sample size is consistent with previous experimental studies evaluating orthosis-related functional outcomes in healthy participants.

## Results

The study included 30 participants evenly distributed between genders (15 females, 15 males) with a mean age of 21 years (SD ± 1.68). The majority of participants were right-handed (86.7%, *n* = 26), while 13.3% (*n* = 4) were left-handed. The mean height was 172.36 cm (SD ± 9.27), mean weight was 63.63 kg (SD ± 10.60), and mean BMI was 21.27 kg/m² (SD ± 1.87) (Table 1).

In the QUEST 2.0 results, both device satisfaction and total scores were significantly higher in the TPU group compared to the PLA group (Device Satisfaction: 4.41 ± 0.60 vs. 3.47 ± 0.62, *p* < 0.001, Kendall’s W = 0.48, Total: 4.60 ± 0.40 vs. 3.98 ± 0.41, *p* < 0.001, Kendall’s W = 0.48). Similarly, the LTT group demonstrated significantly higher scores than the PLA group in both domains (device satisfaction: 3.98 ± 0.41 vs. 3.47 ± 0.62, total: 4.48 ± 0.37 vs. 3.98 ± 0.41; *p* = 0.001). However, no statistically significant differences were observed between the TPU and LTT groups in either domain. Service satisfaction scores showed no statistically significant differences among any of the groups (*p* > 0.05; Table 2).

In the Jebsen Taylor Hand Function Test (JTT), statistically significant differences were observed in specific subtests. TPU demonstrated a statistically significant faster completion time in J2 (cards) compared to PLA (6.40 ± 2.43 s vs. 9.01 ± 2.49 s, *p* < 0.001, Kendall’s W = 0.27). For J3 (small objects), LTT achieved a statistically significant faster completion time than PLA (7.85 ± 3.25 s vs. 11.56 ± 4.82 s, *p* = 0.017, Kendall’s W = 0.13). TPU outperformed LTT in J4 (simulated feeding) with a statistically significant faster completion time (8.69 ± 2.13 s vs. 10.60 ± 2.30 s, *p* < 0.001, Kendall’s W = 0.24). Regarding the total JTT score, TPU achieved the best overall performance, which was statistically significant compared to both PLA (47.91 ± 6.01 s vs. 56.39 ± 6.36 s, *p* < 0.001, Kendall’s W = 0.52) and LTT (47.91 ± 6.01 s vs. 53.38 ± 7.07 s, *p* = 0.002, Kendall’s W = 0.52). For all other subtests, no statistically significant differences were observed among the groups (*p* > 0.05; Table 3).

**Table 1:** Demographic characteristics of the patients (*n* = 30).

Variable		*n* = 30
Gender, *n*	Female	15
	Male	15
Dominant hand, n	Left	4
	Right	26
Age (y), mean (SD)		21 (1.68)
Height (cm), mean (SD)		172.36 (9.27)
Weight (kg), mean (SD)		63.63 (10.60)
BMI (kg/m^2^), mean (SD)		21.27 (1.87)

BMI= Body Mass Index.

SD= standard deviation.

**Table 2:** Comparison of QUEST 2.0 Scores.

QUEST 2.0 (0–5)	Orthosis Material	Mean Score (± SD)	*p*-value
Device satisfaction	TPU (*n* = 30)	4.41 (0.60)	0.001*
	PLA (*n* = 30)	3.47 (0.62)	
	TPU (*n* = 30)	4.41 (0.60)	0.320*
	LTT (*n* = 30)	3.98 (0.41)	
	PLA (*n* = 30)	3.47 (0.62)	0.001*
	LTT (*n* = 30)	3.98 (0.41)	
Service satisfaction	TPU (*n* = 30)	5.00 (0.00)	1.00*
	PLA (*n* = 30)	5.00 (0.00)	
	TPU (*n* = 30)	5.00 (0.00)	1.00*
	LTT (*n* = 30)	5.00 (0.00)	
	PLA (*n* = 30)	5.00 (0.00)	1.00*
	LTT (*n* = 30)	5.00 (0.00)	
Total	TPU (*n* = 30)	4.60 (0.40)	0.001*
	PLA (*n* = 30)	3.98 (0.41)	
	TPU (*n* = 30)	4.60 (0.40)	0.320*
	LTT (*n* = 30)	4.48 (0.37)	
	PLA (*n* = 30)	3.98 (0.41)	0.001*
	LTT (*n* = 30)	4.48 (0.37)	

SD = standard deviation; QUEST 2.0 = Quebec User Evaluation of Satisfaction with Assistive Technology.

TPU= thermoplastic polyurethane, PLA= polylactic acid, LTT = low temperature thermoplastic.

* Overall comparisons among orthoses were performed using the Friedman test. When statistically significant differences were detected, post hoc pairwise comparisons were conducted using the Wilcoxon signed-rank test with Bonferroni correction. Effect sizes (Kendall’s W) are reported in the Results text for significant Friedman tests.

**Table 3:** Comparison of JTT subtest and total scores.

JTT	Orthosis Material	Mean Score (± SD)	*p*-value
J1 writing (s)	TPU (*n* = 30)	13.38 (2.36)	0.082*
	PLA (*n* = 30)	14.77 (2.06)	
	TPU (*n* = 30)	13.38 (2.36)	
	LTT (*n* = 30)	15.08 (2.67)	
	PLA (*n* = 30)	14.77 (2.06)	
	LTT (*n* = 30)	15.08 (2.67)	
J2 cards (s)	TPU (*n* = 30)	6.40 (2.43)	0.001*
	PLA (*n* = 30)	9.01 (2.49)	
	TPU (*n* = 30)	6.40 (2.43)	0.212*
	LTT (*n* = 30)	7.44 (2.46)	
	PLA (*n* = 30)	9.01 (2.49)	0.085*
	LTT (*n* = 30)	7.44 (2.46)	
J3 small objects (s)	TPU (*n* = 30)	8.65 (3.34)	0.413*
	PLA (*n* = 30)	11.56 (4.82)	
	TPU (*n* = 30)	8.65 (3.34)	0.59*
	LTT (*n* = 30)	7.85 (3.25)	
	PLA (*n* = 30)	11.56 (4.82)	0.017*
	LTT (*n* = 30)	7.85 (3.25)	
J4 simulated feeding (s)	TPU (*n* = 30)	8.69 (2.13)	0.212*
	PLA (*n* = 30)	9.84 (2.22)	
	TPU (*n* = 30)	8.69 (2.13)	0.001*
	LTT (*n* = 30)	10.60 (2.30)	
	PLA (*n* = 30)	9.84 (2.22)	0.136*
	LTT (*n* = 30)	10.60 (2.30)	
J5 checkers (s)	TPU (*n* = 30)	3.21 (1.16)	0.331*
	PLA (*n* = 30)	3.10 (0.84)	
	TPU (*n* = 30)	3.21 (1.16)	
	LTT (*n* = 30)	4.04 (2.09)	
	PLA (*n* = 30)	3.10 (0.84)	
	LTT (*n* = 30)	4.04 (2.09)	
J6 large light objects (s)	TPU (*n* = 30)	3.75 (0.84)	0.076*
	PLA (*n* = 30)	4.17 (0.93)	
	TPU (*n* = 30)	3.75 (0.84)	
	LTT (*n* = 30)	3.97 (0.88)	
	PLA (*n* = 30)	4.17 (0.93)	
	LTT (*n* = 30)	3.97 (0.88)	
J7 large heavy objects (s)	TPU (*n* = 30)	3.80 (0.72)	0.344*
	PLA (*n* = 30)	3.94 (0.96)	
	TPU (*n* = 30)	3.80 (0.72)	
	LTT (*n* = 30)	4.39 (1.16)	
	PLA (*n* = 30)	3.94 (0.96)	
	LTT (*n* = 30)	4.39 (1.16)	
Total JTT score (s)	TPU (*n* = 30)	47.91 (6.01)	0.001*
	PLA (*n* = 30)	56.39 (6.36)	
	TPU (*n* = 30)	47.91 (6.01)	0.002*
	LTT (*n* = 30)	53.38 (7.07)	
	PLA (*n* = 30)	56.39 (6.36)	0.213*
	LTT (*n* = 30)	53.38 (7.07)	

SD = standard deviation; JTT = Jebsen Taylor Hand Function Test.

TPU= thermoplastic polyurethane, PLA= polylactic acid, LTT = low temperature thermoplastic.

*Overall comparisons among orthoses were performed using the Friedman test. When statistically significant differences were detected, post hoc pairwise comparisons were conducted using the Wilcoxon signed-rank test with Bonferroni correction. Effect sizes (Kendall’s W) are reported in the Results text for significant Friedman tests.

## Discussion

This study provides insights into the hand function and user satisfaction associated with different orthosis materials used in CMC joint stabilization orthoses. To our knowledge, this is one of the first studies to comprehensively compare TPU, PLA, and LTT in this context, highlighting material-related differences in subjective and objective outcomes under controlled experimental conditions. While studies exist on the production and effectiveness of 3D-printed hand orthoses, research comparing different filament types used in 3D-printed orthoses is very limited. Specifically, no studies have been conducted on filament comparisons in CMC joint stabilization orthoses.

According to QUEST 2.0 survey results, TPU and LTT demonstrated statistically significantly higher user satisfaction scores compared to PLA. These results suggest that these two materials were associated with higher perceived user satisfaction during short-term use. The lack of a statistically significant difference in satisfaction scores between TPU and LTT indicates that these two materials meet user expectations at a comparable level. TPU’s material properties, especially flexibility and durability^14^, may positively contribute to user satisfaction. These material properties may contribute to differences in perceived comfort and task performance during experimental testing.

Functional outcomes, as assessed through the JTT, support TPU’s potential advantages in this context. In J2 (cards), TPU demonstrated statistically significantly faster completion times compared to PLA. In J3 (small objects), LTT outperformed PLA, reflecting its suitability for tasks requiring precision and grip. In J4 (simulated feeding), TPU was statistically significantly faster than LTT, highlighting its potential for enhancing fine motor tasks. For total JTT scores, TPU completed tasks statistically significantly faster than both PLA and LTT, indicating material-related differences in task performance during standardized functional testing. The observed differences in JTT performance may be related to material flexibility and compliance, which can influence movement during task execution. The functional differences observed between orthosis materials may be partially explained by their distinct mechanical properties. TPU is characterized by a lower modulus of elasticity and greater flexibility compared with PLA and LTT. This increased compliance allows TPU to deform slightly during movement while maintaining elastic recovery, potentially facilitating smoother thumb motion during dynamic tasks. In contrast, the higher stiffness and rigidity of PLA may restrict adaptive movement, while LTT, although moldable, may exhibit reduced flexibility once cooled. In addition, differences in surface characteristics and friction at the skin–orthosis interface may influence comfort and movement efficiency, particularly during repetitive or fine motor tasks. These material-specific properties may contribute to the observed differences in task performance under experimental conditions^14,15^.

Although the present study did not investigate clinical outcomes in symptomatic populations, clinical studies have shown promising results regarding the effectiveness of 3D-printed orthoses in improving functional outcomes. For example, a randomized controlled trial indicated that patients using 3D-printed orthoses experienced significant improvements in wrist flexor spasticity compared to those using conventional thermoplastic orthoses^19^. Additionally, the aesthetic appeal of 3D-printed devices has been highlighted as a factor that can enhance patient satisfaction, as users often prefer devices that are visually appealing and less bulky^20^.

The effectiveness of 3D-printed orthoses, particularly for hand rehabilitation, has garnered increasing attention in recent years. Research indicates that 3D-printed orthoses can be effective alternatives to conventional orthotic devices. For instance, one randomized controlled trial (RCT) demonstrated that patients using 3D-printed orthoses reported significantly better outcomes in terms of pain, range of motion (ROM), and overall hand function compared to those with conventional orthoses^21^. Moreover, the customization capabilities of 3D printing allow for the creation of orthoses that are tailored to the individual anatomical features of patients, which can enhance comfort and compliance. For example, a study on the production of wrist-hand orthoses noted that the use of 3D printing technology resulted in devices that were not only aesthetically pleasing but also more comfortable for the wearer, which is crucial for long-term use^20^.

Research on orthoses for patients with peripheral nerve injuries indicated that 3D-printed devices could lead to substantial improvements in hand function^22^.

In a study conducted by Kim et al. (2018), a comparison between 3D-printed TPU and ready-made volar cock-up orthoses revealed that in the JTT simulated feeding subtest, ready-made orthoses demonstrated statistically significantly faster completion times than 3D-printed TPU. No differences were observed between orthoses in other comparisons. In contrast, our findings showed that 3D-printed TPU achieved statistically significantly faster times than LTT orthoses in simulated feeding. Additionally, TPU had the shortest total time compared to the other materials. However, in the Kim et al. (2018) study, no differences were found in other comparisons between ready-made and 3D-printed TPU orthoses. Although comparing studies with different materials and orthoses may not be appropriate, the functional and satisfaction outcomes of 3D-printed orthoses being similar to ready-made orthoses in Kim et al.‘s study may offer contextual insight, although differences in orthosis type, materials, and study populations limit direct comparison^23^.

The cost of 3D-printed orthoses varies depending on the type of material used, the complexity of the design, and the production process. For example, Popescu et al. (2020) reported that the total production time for 3D-printed hand orthoses was 23.7 h, with 6 h spent on the design phase^12^. In our study, the production time was 80 min, and the design phase took 20 min, highlighting the variability in processes depending on the software, 3D printer, and individual skills involved. This variability may also stem from the novelty of the technology and the limited number of professionals currently using it in orthotic applications. We anticipate that as this technology becomes more widespread in clinical settings and as orthosis-specific software programs proliferate, such differences will diminish. From a clinical perspective, production time is a critical determinant of feasibility, particularly in high-volume outpatient settings. The production time observed in the present study (approximately 80 min, including design and printing) suggests that 3D-printed CMC orthoses could be realistically integrated into routine clinical workflows, potentially allowing same-day or next-day orthosis delivery. In contrast, substantially longer production times reported in previous studies may limit applicability in busy clinical environments. Standardization of orthosis design templates, user-friendly software platforms, and targeted clinician training may further reduce production time and variability, facilitating broader implementation of 3D printing technology in everyday practice.

Additionally, 3D printing technology enables the production of lighter and more durable orthoses with reduced material consumption^15^. In terms of cost, the initial costs of 3D-printed orthoses may be higher compared to traditional plaster or plastic orthoses; however, these costs are expected to decrease in the long term. For instance, Graham et al. (2018) highlighted the advantages of 3D-printed orthoses in terms of comfort and fit compared to conventional methods^24^. Moreover, 3D-printed orthoses have been shown to help patients achieve better outcomes in their treatment processes^20^. In our study, the observed material-related differences in user satisfaction and task performance are consistent with the broader literature highlighting the potential of 3D-printed orthoses, although direct clinical comparison is not appropriate.

The production of hand orthoses using 3D printing stands out with its cost-effectiveness (disregarding high initial costs), customization potential, and production time advantages. However, further research and development are needed to reduce initial costs and promote the widespread adoption of this technology. The potential of 3D printing technology warrants further investigation in orthopedic rehabilitation, particularly through clinically focused studies^25,26^.

The use of healthy participants enabled isolation of material-specific effects; however, it also limits direct clinical generalization to symptomatic CMC osteoarthritis populations. According to the results, TPU orthosis has slightly better results than other orthoses in terms of functionality and satisfaction. PLA orthosis received the lowest score in terms of satisfaction. Orthoses produced with 3D printers generally had similar results to LTT orthoses. Within the limits of this experimental design, TPU orthoses demonstrated higher satisfaction scores and faster task completion in selected measures. Further studies need to be investigated in terms of the flexibility of the TPU material as well as the production technique of CMC joint stabilization orthosis.

Limitations: Despite these promising results, this study has limitations. The relatively small sample size (*n* = 30) may constrain the generalizability of the findings, and the study focused only on short-term outcomes. Long-term wearability and durability of the orthoses were not assessed, leaving a gap for future research. Moreover, the lack of a non-intervention control group limits the ability to fully isolate the effects of material differences. Therefore, the findings should not be interpreted as evidence of clinical effectiveness in individuals with CMC osteoarthritis. Each orthosis was worn for a relatively short period (two hours), which limits conclusions regarding long-term comfort, durability, wearability, and real-world use. Although the order of orthosis testing was randomized, the short testing period may have introduced order-related or learning effects. The absence of participant blinding represents another limitation, particularly for subjective satisfaction outcomes. Furthermore, important factors related to long-term adherence—such as breathability, ease of cleaning, and material durability, particularly when comparing TPU with traditional low-temperature thermoplastics—could not be evaluated within the short-term experimental design of this study. Future studies with extended wear periods are needed to address these aspects.

### Conclusion

This study focused on the effects of 3D (TPU, PLA) and LTT orthoses on satisfaction and hand function in healthy individuals. The findings provide preliminary, material-specific information that may inform future research involving symptomatic CMC joint conditions. Within the limits of short-term testing, TPU demonstrated higher user satisfaction scores and faster task performance in selected measures compared with PLA and LTT. While LTT demonstrated comparable performance in some measures, the observed material-related differences warrant further investigation rather than direct clinical recommendation. Further clinical research with longer follow-up periods and symptomatic CMC populations is required to determine the clinical relevance of these findings.

## Data Availability

The data supporting the findings of this study are available upon reasonable request. Due to the nature of the study, the raw data cannot be publicly shared to protect participant confidentiality. However, the minimal dataset necessary to interpret, replicate, and build upon the findings reported in this article can be provided upon request from the corresponding author.If applicable, data may also be shared in anonymized form through a suitable repository, or as supplementary materials, upon acceptance and publication of the article.
